# Virtual Balancing for Studying and Training Postural Control

**DOI:** 10.3389/fnins.2017.00531

**Published:** 2017-09-26

**Authors:** Daniela Buettner, Daniela Dalin, Isabella K. Wiesmeier, Christoph Maurer

**Affiliations:** Department of Neurology and Neurophysiology, University Hospital Freiburg, Medical Faculty, Freiburg, Germany

**Keywords:** virtual, postural control, balancing, inverted pendulum, model

## Abstract

Postural control during free stance has been frequently interpreted in terms of balancing an inverted pendulum. This even holds, if subjects do not balance their own, but an external body weight. We introduce here a virtual balancing apparatus, which produces torque in the ankle joint as a function of ankle angle resembling the gravity and inertial effects of free standing. As a first aim of this study, we systematically modified gravity, damping, and inertia to examine its effect on postural control beyond the physical constraints given in the real world. As a second aim, we compared virtual balancing to free stance to test its suitability for balance training in patients who are not able to balance their full body weight due to certain medical conditions. In a feasibility study, we analyzed postural control during free stance and virtual balancing in 15 healthy subjects. Postural control was characterized by spontaneous sway measures and measures of perturbed stance. During free stance, perturbations were induced by pseudorandom anterior-posterior tilts of the body support surface. In the virtual balancing task, we systematically varied the anterior-posterior position of the foot plate where the balancing forces are zero following a similar pseudorandom stimulus profile. We found that subjects' behavior during virtual balancing resembles free stance on a tilting platform. This specifically holds for the profile of body excursions as a function of stimulus frequencies. Moreover, non-linearity between stimulus and response amplitude is similar in free and virtual balancing. The overall larger stimulus induced body excursions together with an altered phase behavior between stimulus and response could be in part explained by the limited use of vestibular and visual feedback in our experimental setting. Varying gravity or damping significantly affected postural behavior. Inertia as an isolated factor had a mild effect on the response functions. We conclude that virtual balancing may be well suited to simulate conditions which could otherwise only be realized in space experiments or during parabolic flights. Further studies are needed to examine patients' potential benefit of virtual balance training.

## Introduction

Free stance is controlled by the central nervous system (CNS) using sensory signals derived from visual, vestibular and proprioceptive afferent information (Dichgans et al., 1976; Lestienne et al., 1976; Nashner and Berthoz, 1978; Freyler et al., 2014; Ritzmann et al., 2015). Stabilization of the center of mass of the human body resembles balancing an inverted pendulum (Ritzmann et al., 2015). Muscle forces are used to provide an appropriate torque (Dichgans et al., 1976), which counteracts gravitational and inertial forces.

The ability to stand freely is often characterized by analyzing spontaneous sway (Prieto et al., 1996; Qu et al., 2009). Spontaneous sway reflects the small fluctuations of body position during standing. These fluctuations show similar patterns in free stance as well as when balancing an external weight through a moveable platform (Fitzpatrick et al., 1992; Fitzpatrick and McCloskey, 1994; Loram and Lakie, 2002). The similarity covers many features of spontaneous sway, like, e.g., amplitude, velocity, and major frequency content. The diagnostic value of spontaneous sway for evaluating stance behavior has been repeatedly questioned. In contrast, studying postural reactions to external perturbations seem to provide a deeper inside into the mechanisms of stance (Peterka, 2002; Maurer and Peterka, 2005; Masani et al., 2006; Lockhart and Ting, 2007; Welch and Ting, 2009; Vette et al., 2010; Davidson et al., 2011; van der Kooij and Peterka, 2011; Nishihori et al., 2012; Engelhart et al., 2014; Pasma et al., 2014). However, whether postural reactions subsequent to external perturbations are similar between free standing and balancing an external weight, is unknown as yet.

The virtual balance paradigm introduced here allows for analyzing both, spontaneous fluctuations and motor reactions to external perturbations. Instead of a physical weight which is balanced through a foot plate, we simulated balancing a weight through a foot plate by measuring the platform to body position, i.e., the ankle joint angle, and calculating the appropriate torque signal, which is then fed back into the system as an ankle torque. We hypothesize that free standing postural reactions could be well mimicked by a virtual balance paradigm after optimizing control parameters.

An important feature of the virtual balance paradigm relates to the nearly free choice of gravitational load, body inertia, and joint damping. From literature, it is well known that postural control is load-dependent (Freyler et al., 2014; Ritzmann et al., 2015). Earlier studies dealing with over- and under-loading were performed with astronauts in space (Layne et al., 2001; Loomer, 2001; di Prampero and Narici, 2003), in free fall conditions (Nomura et al., 2001; Miyoshi et al., 2003), with partial weight-bearing (Ali and Sabbahi, 2000; Phadke et al., 2006; Hwang et al., 2011; Freyler et al., 2014), under hypergravity (Miyoshi et al., 2003), with extra weight (Dietz et al., 1989; Ali and Sabbahi, 2000), or using water buoyancy (Dietz et al., 1989; Nakazawa et al., 2004). It has been demonstrated that load variation is associated with changes in angle torque (Mergner and Rosemeier, 1998; Nakazawa et al., 2004), in the use of somatosensory signals (Paloski et al., 1993; Bloomberg et al., 1997; Layne et al., 2001), and changes in neuromuscular activity (Dietz et al., 1989; Avela et al., 1994; Ali and Sabbahi, 2000; Pöyhönen and Avela, 2002). However, despite the substantial amount of load-related articles, the underlying neuromuscular mechanisms and functional consequences for balance control are poorly understood.

The paradigm of modifying gravitational load and inertia seems to be particularly helpful in patients who are not capable of supporting their own body weight, e.g., due to trauma or muscle weakness. It is well known that neural mechanisms employed to perform balancing are different from those used to, e.g., apply ankle torque against an external resistance, including the different sensory perceptions (Fitzpatrick and McCloskey, 1994). Here, we aimed to examine the individual effects of gravity, damping and inertia on postural control that could otherwise only be realized in space experiments or during parabolic flights. Moreover, we aimed at comparing virtual balancing with free stance to investigate its similarity and suitability for balance training in patients who are not able to balance their full body weight and/ or are prone to falls. Finally, virtual balancing may allow for adapting gravitational load and inertia to patients' needs.

## Materials and methods

In this feasibility study, subjects were tested by recording spontaneous sway, as well as motor reactions to external perturbations during free stance, and, in addition, using a virtual balance apparatus.

### Subjects

We measured postural control of 15 young people (9 female, 6 male, 23.8 years ± 2.14 [mean age ± SD]). We excluded people suffering from any disease that may interact with postural control. For that, each subject was carefully examined for intact vestibular and proprioceptive function. Further exclusion criteria included any acute or chronic disease that may influence the general condition of health. Anthropometric data of subjects are given in Table 1.

**Table 1:** Anthropometric data of subjects.

**Subject**	**Height (cm)**	**Weight (kg)**	**BMI (kg/m^2^)**
1	172	57	19.3
2	180	71	21.9
3	174	73	24.1
4	174	70	23.1
5	173	68	22.7
6	183	70	20.9
7	197	76	19.6
8	182	80	24.2
9	180	70	21.6
10	158	58	23.2
11	160	45	17.6
12	160	51	19.9
13	157	48	19.5
14	163	55	20.7
15	164	71	26.4
Mean value ± SD	171.8 ± 11.06	64.2 ± 9.77	21.7 ± 2.23

*9 female, 6 male; SD, standard deviation; BMI, body mass index; cm, centimeter; kg, kilogram; m, meter*.

### Procedures during free standing

**Figure 1:**
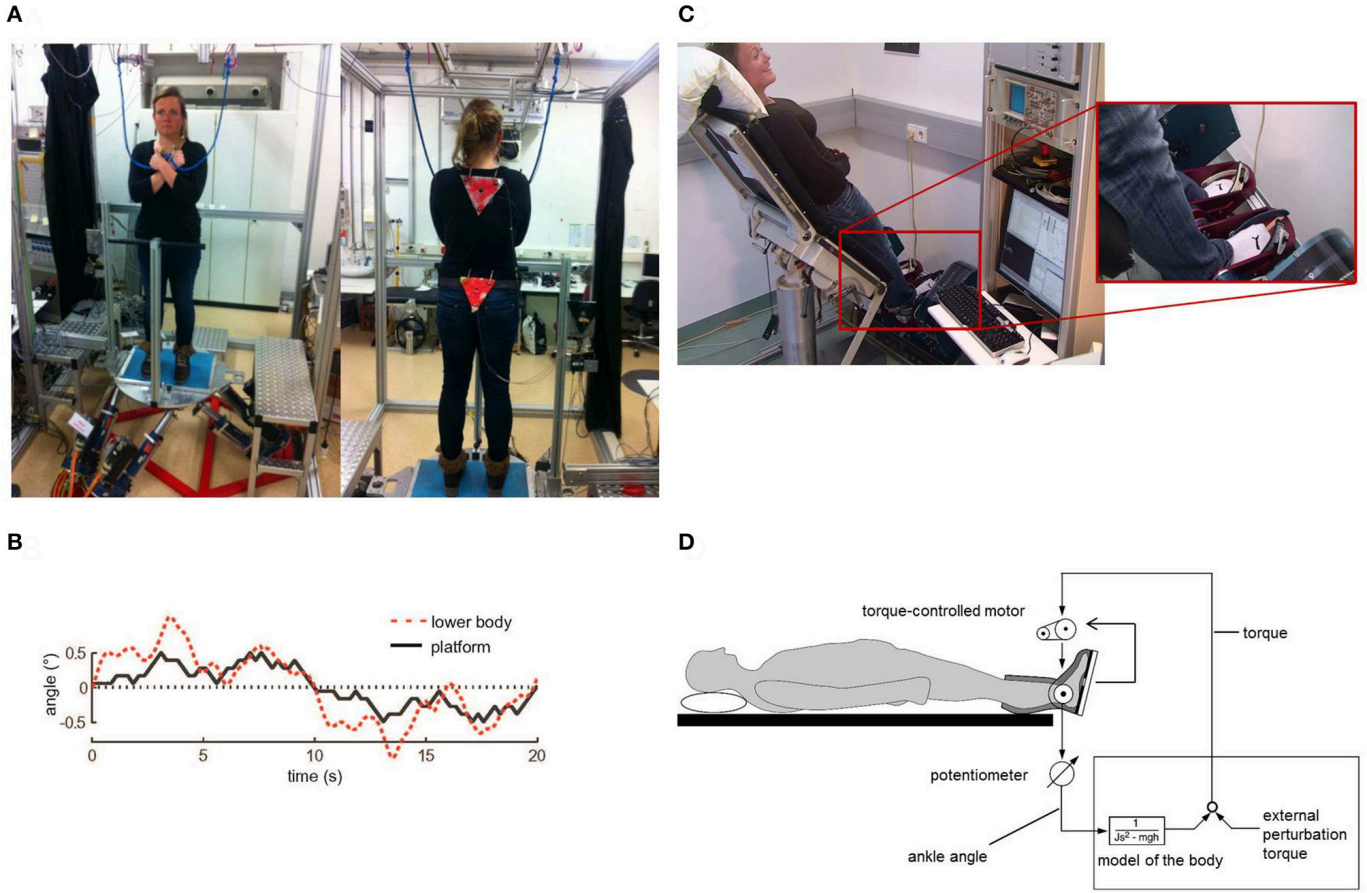
Experimental setup. **(A)** Custom-built motion platform for measurements of spontaneous sway and perturbed stance. Angular excursions of the body and the platform were quantified with an optoelectronic camera system. **(B)** Example of a PRTS (pseudorandom ternary sequence) stimulus profile (black line) yielding to anterior-posterior rotational tilts of the platform with 1° peak-to-peak stimulus amplitude together with a healthy subject's postural reaction in terms of lower body excursion (red dotted line). **(C)** Custom-made platform for virtual balancing. Subjects were lying in an inclined position with their feet positioned on a tilting balance board. They moved their feet in the ankle joint in order to control the balance board. **(D)** Potentiometers and torque sensors measured position as well as torque of the balance board with respect to the body. Measured board tilts were transferred online into board torque commands using the real time simulation toolbox of Simulink/Matlab, running on a PC. The ankle angle-to-torque transformation followed the characteristics of an inverted pendulum body, including virtual gravity (mgh), inertia (J) and damping. Board torque commands were fed back to the board via a torque controlled electric motor. External perturbations, i.e., PRTS stimuli, were applied as shifts of the zero equilibrium point.

Spontaneous sway and perturbed stance were assessed on a custom-built motion platform (Figure 1A). Subjects were told to stand upright in a relaxed position. For safety reasons and to prevent falls, the experimental setup included ropes that were fixed to the ceiling at a position which was about 30 cm in forward direction with respect to the foot position of the subjects. At the lower end of the ropes, there were two small wooden handles attached to the rope. The handles did not serve as a reference, because the subjects held them freely. Since they were attached via the loosely dangling ropes to the ceiling, they were meant to serve as a replacement for a body harness. If a subject felt unsafe, he/ she could have lowered the handles to put tension on the ropes. However, this event did not happen during the experiments. None of the subjects used the ropes to prevent falls. Subjects performed 2 trials of spontaneous sway and 8 trials of perturbed stance, each with eyes open and eyes closed. One trial lasted 1 min; between trials, a short break of about 10 s was taken.

We recorded center-of-pressure (COP) sway paths and 3-D angular positions of the body. The COP sway path was measured with the help of a force transducing platform (Kistler platform type 9286, Winterthur, Switzerland). 3-D angular excursions of the body (hip-to-ankle, shoulder-to-hip) were detected using an optoelectronic device with markers attached to shoulder and hip (Optotrak 3020, Waterloo, Canada). Each marker consisted of three light-emitting diodes fixed to a rigid triangle. Optotrak® and Kistler® output signals as well as the stimulus signals recorded with software programmed in LabView® (National Instruments, Austin, Texas, USA). COM height above the ankle joints was calculated according to tables from Winter (1995) using the measured heights of hip and shoulder markers. A detailed description of the experimental setup has been published previously (e.g., Wiesmeier et al., 2015).

External perturbations consisted of rotational platform tilts in the sagittal plane with the tilt axis passing through subject's ankle joints. Stimulus profiles followed a pseudorandom signal (PRTS, pseudorandom ternary sequence, see Figure 1B) using two different peak-to-peak amplitudes (0.5° and 1°). Postural reactions were evaluated at certain frequencies (0.05, 0.15, 0.3, 0.4, 0.55, 0.7, 0.9, 1.1, 1.35, 1.75, and 2.2 Hz), leading to specific transfer functions between stimulus and postural reactions.

### Procedures during virtual balancing

Virtual balancing was performed on a newly developed, custom-made platform. Subjects were lying in an inclined position with their feet positioned on a tilting board (Figure 1C). In this experimental setting, subjects did not move their own body. They solely moved their feet in the ankle joint in order to control the moveable balance board (Figure 1D). We measured position as well as torque of the balance board with respect to the body using potentiometers and torque sensors. The balance board was programed in such a way that its anterior-posterior torque was derived from its position. Similar to the gravitational effect in a free standing task, the virtual gravitational torque had a zero point at a board position orthogonal to the body vertical direction. Increasing board angles were accompanied by increasing torques away from the zero position, resembling unstable equilibrium. The inertial force was mimicked through an additional torque which was directed counter to the board acceleration. Damping was implemented by applying a counter-torque based on board velocity. Virtual gravity, inertia and damping were calculated with a compiled version of a Simulink/MATLAB® model (The MathWorks Inc., Natick, MA, USA) using the real-time simulation mode. Measured board tilts were transferred online into board torque commands, which were then fed back to the board via torque controlled electric motors. This condition resembled free standing on firm ground and produced spontaneous sway.

In the virtual external perturbation condition, we used a stimulus profile similar to free standing (PRTS, see above). This signal directly modified the zero-position of the equilibrium. The stimulus produced an additional torque. For example, if we aimed to simulate a one degree tilt of the platform during free standing, we added a torque with a size exactly resembling the gravitational torque of a one degree inclination from space vertical of a human body. Subjects tended to correct for this torque offset by moving the platform until a new equilibrium with minimal torque was found. This behavior is related to the free standing behavior on a tilting platform, where a platform tilt leads to a correction of the ankle angle until the body is close to vertical in space again, thereby minimizing gravitational torque.

Subjects were instructed to comfortably lean on the inclined backboard and fold their arms. We assured that no relevant body movements were elicited other than subjects' ankle joint movements controlling the balance board. Subjects were instructed to continuously balance the moveable platform and find the equilibrium point in a playful manner rather than to massively co-contract in order to block the platform in a certain position. Subjects were presented with 5 trials for practice. Subsequently, we systematically varied gravitational force of the virtual body (10, 20, and 40% of subjects' own body weight), inertial force (5, 10, and 20% of subjects' own body inertia), damping (5 and 10% of the gravitational force), and external perturbation (relating to 0, 0.25°, 0.5°, and 1° peak to peak body inclination, with respect to subjects' body metrics). The experimental protocol consisted of 3 × 3 × 2 × 4 = 72 trials in a randomized order. Each trial started with a 10 s period, where the torque with respect to the board position was ramping up to allow subjects for finding their equilibrium point. The duration and the stimulus profile of the subsequent measuring period closely resembled the trials during free standing.

### Data analyses

Data analysis was performed off-line with custom-made software programmed in MATLAB® (The MathWorks Inc., Natick, MA, USA). From upper body, lower body and center of pressure excursions in the free standing condition and board tilts in the virtual balancing condition, we calculated Root Mean Square (sway amplitude, RMS) and Mean velocity (sway velocity, MV) for characterizing spontaneous sway. Transfer functions from stimulus-response data were calculated by a discrete Fourier transform. Fourier coefficients of stimulus and response time series are used to determine GAIN and PHASE with respect to stimulus frequencies. GAIN (response sensitivity) shows the amplitude relationship between the external perturbation and the postural reaction (body angle during free standing, board angle during virtual balancing). PHASE is the relative delay between the stimulus and the reaction of the body.

Statistical analyses were performed using Microsoft Excel and statistic programs (JMP® and Statview by SAS Institute Inc., Cary, NC, USA). After testing normal distribution and homogeneity of variances with the Kolmogorov-Smirnov test, we used parametric methods for further analyses. Due to the expected dependency between the outcome measures within the balance tasks (real stance vs. virtual balancing), statistical significance was tested by an analysis of variance (ANOVA). The within-subjects factors for free spontaneous sway were visual condition, sway direction, and body segment (hip, shoulder). For perturbed stance, the within-subjects factors were visual condition, stimulus amplitude, stimulus frequency, and body segment (hip, shoulder). For the virtual balancing task, the within-subjects factors were gravity, damping, inertia, and stimulus amplitude. The level of statistical significance was set at *p* = 0.05.

The study was performed according to the ethical standards of the Declaration of Helsinki. It was approved by the ethics committee of the Medical Center of the University of Freiburg. All subjects gave their written informed consent prior to study participation.

## Results

### Spontaneous sway

**Figure 2:**
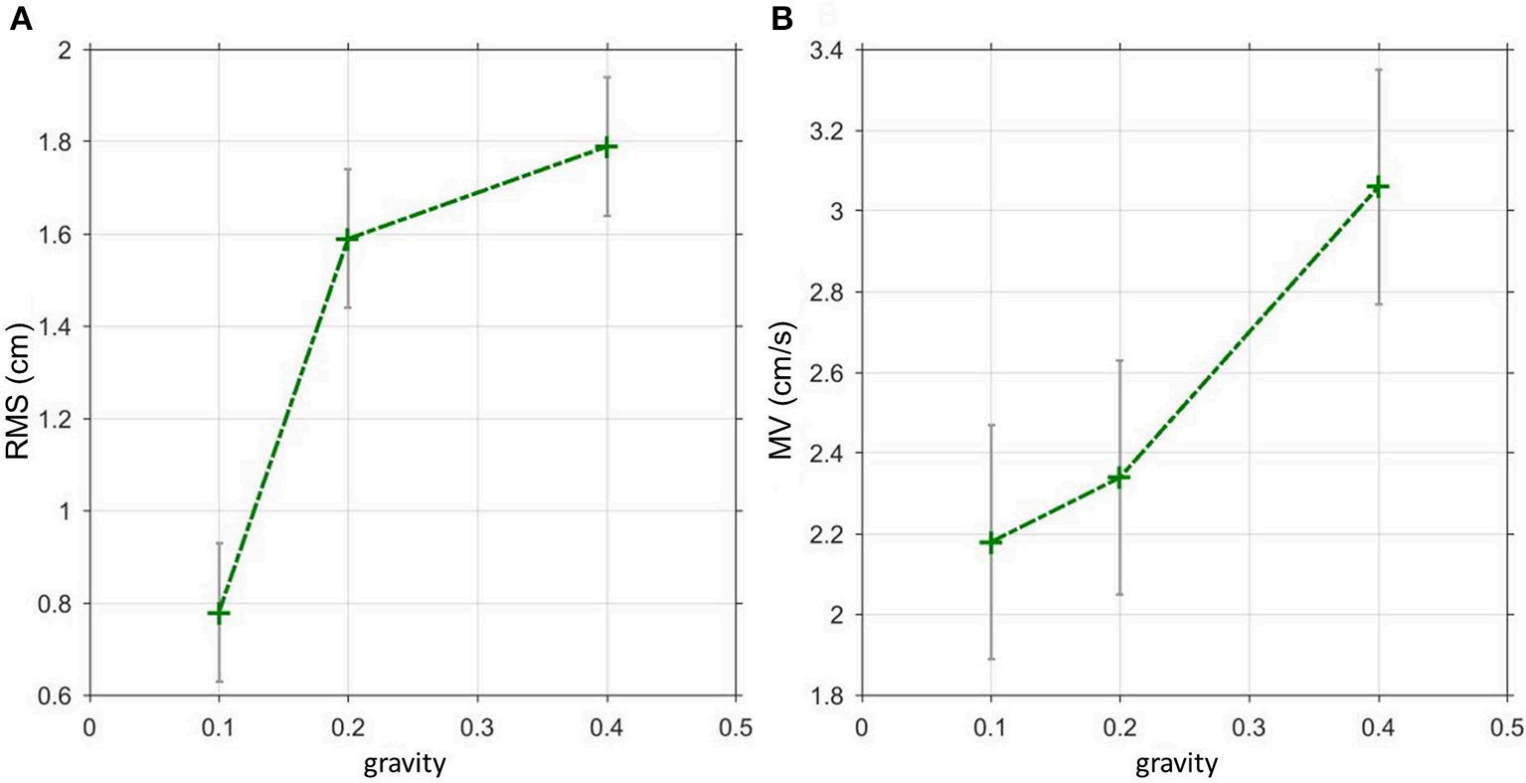
Spontaneous sway during virtual balancing. **(A)** Root Mean Square (RMS) and **(B)** Mean Velocity (MV) as a function of the amount of virtual gravity.

Generally, the Root Mean Square (sway amplitude, RMS) of virtual balancing (3.15 cm) was significantly larger than the RMS of free stance (0.47 cm, eyes open). Moreover, the RMS during virtual balancing significantly depended on gravity (3.15 cm with a gravity effect of 40% vs. 2.80 cm with a gravity effect of 20% and 1.37 cm with 10%; *F* = 23.04, *p* < 0.0001, Figure 2A). In contrast, inertia and damping did not affect RMS. As with RMS, Mean Velocity (sway velocity, MV) of virtual balancing (5.39 cm/s) was significantly larger than the MV of free stance (0.43 cm/s; eyes open) and significantly depended on gravity (5.39 cm/s with a gravity effect of 40% vs. 4.12 cm/s with a gravity effect of 20% and 3.84 cm/s with 10%; *F* = 5.09, *p* = 0.0068, Figure 2B). Inertia and Damping did not significantly affect MV.

### Externally perturbed stance

**Figure 3:**
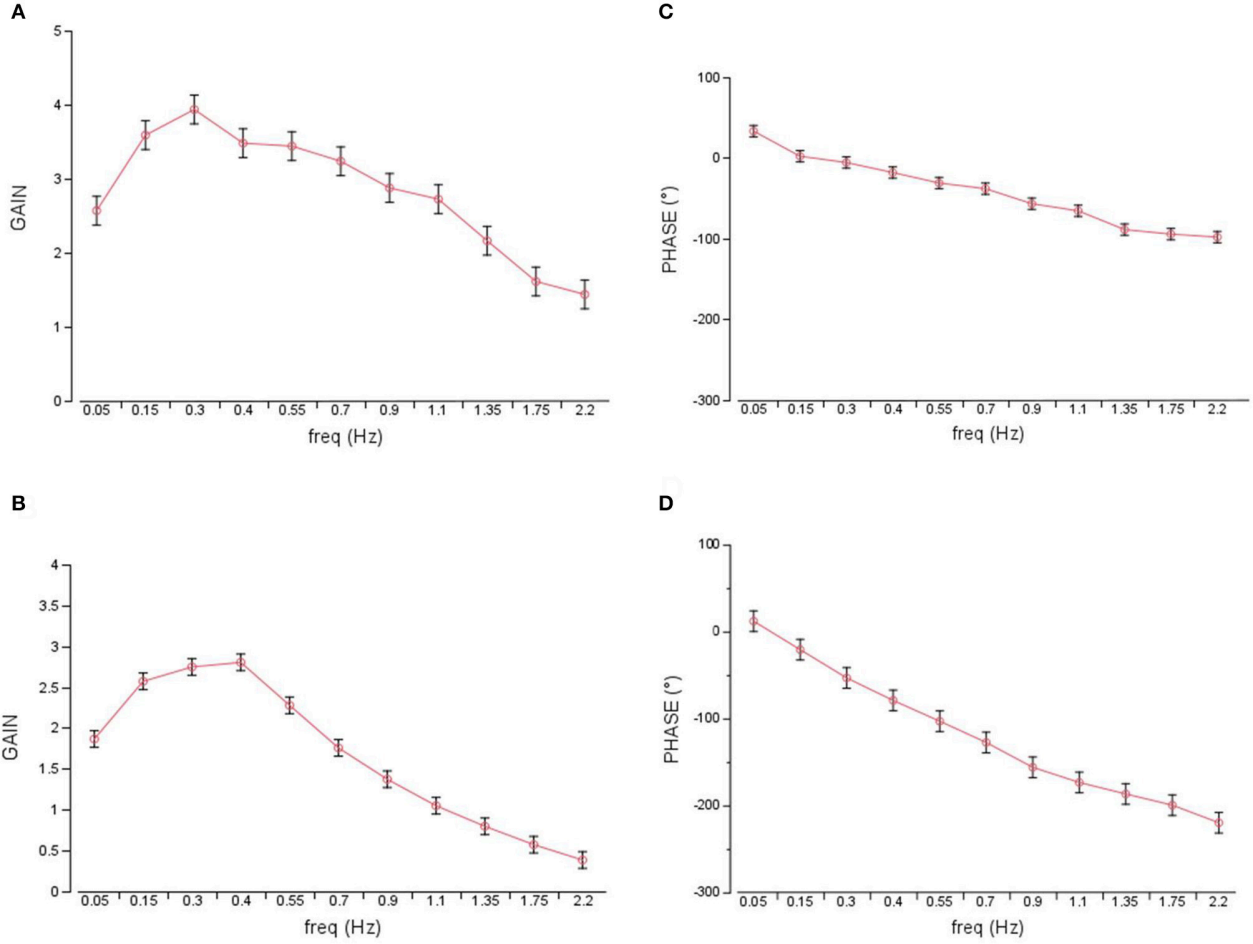
GAIN and PHASE curves representing transfer functions of virtual balancing and free stance. GAIN during virtual balancing **(A)**, and during free stance **(B)** across all parameter settings, freq, frequency in Hz. Respective PHASE (in degree) during virtual balancing **(C)** and during free stance **(D)**.

**Figure 4:**
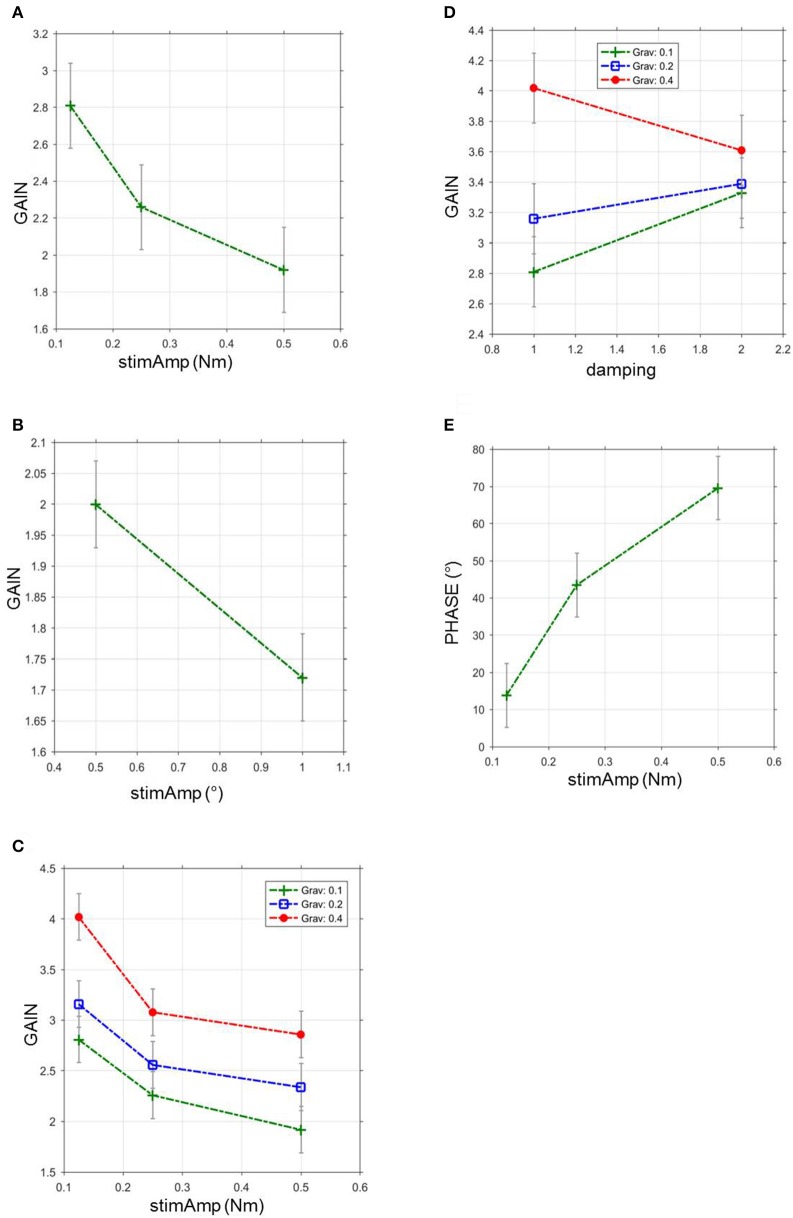
GAIN and PHASE in relation to stimulus amplitude, gravitational force and damping during virtual balancing and free stance. **(A)** GAIN during virtual balancing as a function of stimulus amplitudes in Nm across all visual conditions and body segments, *stimAmp*, stimulus amplitude. **(B)** GAIN during free stance as a function of stimulus amplitudes in degrees across all visual conditions and body segments, *stimAmp*, stimulus amplitude. °, degree. **(C)** Influence of gravitational force and stimulus amplitude on GAIN during virtual balancing, *Grav*, gravitational force **(D)** Influence of gravitational force and damping on GAIN during virtual balancing. **(E)** Relation between stimulus amplitude and PHASE during virtual balancing.

The GAIN across all parameter settings and frequencies during virtual balancing (1.92, Figure 3A) was slightly larger than the GAIN of free stance (1.74, eyes open, Figure 3B). However, the difference between the two experimental sets was much smaller than that for spontaneous sway. The frequency influenced GAIN significantly during virtual balancing (*F* = 19.2, *p* < 0.001). Both virtual balancing and free stance showed the largest GAIN (>3.5) between a frequency of 0.15–0.55 Hz. With increasing frequency, GAIN values decreased. Interactions of stimulus amplitude and GAIN during virtual balancing (2.81 with 0.125 Nm and 1.92 with 0.5 Nm) and free stance (2 with 0.5° and 1.73 with 1°) were similar and indicated a non-linearity between stimulus amplitude and GAIN (Figure 4A virtual balancing, Figure 4B free stance). In the frequency-response curve the Phase showed a similar behavior during virtual balancing and free stance. Low frequencies induced low delay of phase while high frequencies induced a large delay of phase (Figure 3C virtual balancing, Figure 3D free stance). The coherence across all effects was slightly larger during virtual balancing (1.0) than during free stance (0.79).

### Dependence of motor behavior from gravity, damping, inertia, and stimulus amplitude

Stimulus amplitude, virtual gravity, and virtual damping significantly interacted with GAIN of the transfer function. Stimulus amplitudes were inversely correlated with GAIN (*F* = 7.98, *p* = 0.0003, Figure 4A). The largest stimulus (0.5 Nm) induced the least GAIN (1.92 vs. 2.26 with 0.25 Nm and 2.81 with 0.125 Nm). In contrast, the virtual gravitational force was directly correlated with GAIN (*F* = 15.7, *p* < 0.0001, Figure 4C). GAIN with a gravity of 40% (4.02) was larger than the GAIN with a gravity of 20% (3.16) and the GAIN with a gravity of 10% (2.81). A larger amount of damping [10%] diminished the GAIN (3.33; vs. 2.81 with smaller damping [5%]; *F* = 7.64, *p* = 0.0057, Figure 4D). Virtual inertia did not affect GAIN in a significant way (*F* = 2.86, *p* = 0.058).

All parameters, except from damping (*F* = 1.85; *p* = 0.1733), significantly interacted with PHASE of the transfer function. There was no linearity between PHASE and gravity (6.64° with gravity 0.4; 32.15° with gravity 0.2 and 13.87° with gravity 0.1; *F* = 5.21, *p* = 0.0055) and inertia (16.44° with 0.0003; −4.70° with 0.00015 and 13.87 with 0.000075; *F* = 3.40, *p* = 0.0184). In contrast the largest stimulus (0.5 Nm) induced the largest PHASE (69.56°) vs. 43.56° with 0.25 Nm and 13.87° with 0.125 Nm (*F* = 23.4; *p* < 0.0001, Figure 4E).

When adjusting stimulus amplitude, virtual gravity, virtual damping, and virtual inertia, to optimally resemble postural reactions of free standing, we isolated a parameter set of a large stimulus amplitude, low gravity and large damping values.

### Individual perception

Subjects valued each stimulus in terms of how much it resembles free standing. A scale 0 (close resemblance), 1 (indifferent), 2 (no resemblance) was given. Both stimulus amplitude and gravity significantly influenced the perception of resemblance to free standing (stimulus amplitude: *F* = 56.0, *p* < 0.0001, gravity: *F* = 31.44, *p* < 0.0001). The smaller a stimulus the higher the resemblance to free standing (0.13 with 0 Nm, 0.23 with 0.125 Nm, 0.44 with 0.25 Nm and 0.85 with 0.5 Nm). Interactions of gravity and resemblance to free standing during virtual balancing were proportional (0.13 with 0.1 cm/s, 0.3 with 0.2 cm/s and 0.54 with 0.4 cm/s;). Damping and inertia did not influence perception to a significant degree.

## Discussion

We introduced here a virtual balancing paradigm, which calculates and delivers ankle torque in a real-time fashion. The applied torque directly depends on ankle angle position, velocity, and acceleration in a way that it resembles gravity and inertial effects of free standing. Balancing an external weight as a substitute for the own body has been evaluated before (Fitzpatrick and McCloskey, 1994; Loram and Lakie, 2002). Here, subjects did not balance themselves, or an external physical weight, but instead a virtual mass that was adapted to the biomechanical characteristics of the subjects in terms of body height and weight. By using the virtual balancing task, we were able to systematically and independently modify gravity, damping, and inertia, which are usually linked together in the real world. This enabled us to study their individual effects on postural control. Moreover, the modification of gravity, damping, and inertia may be used to train patients who are not able to stabilize their own body. First, we evaluated the effects of gravity, damping, and inertia on postural control. This will be presented in the following paragraphs. As a second aim, we compared virtual balancing to free stance to test its similarity and suitability for balance training in patients who are not able to balance their full body weight due to certain medical conditions. We will discuss that thereafter.

Spontaneous sway parameters clearly depended on virtual gravity. Sway amplitude (RMS) as well as sway velocity (MV) increased with increasing virtual gravity. These findings nicely reproduce results from Ritzmann et al. (2015), where overloading increased, and underloading decreased, postural sway amplitudes and frequencies. This was interpreted in part by a predominance of the ankle strategy as compared to hip strategy to organize postural control with increasing loads (Dietz et al., 1980). However, our experimental setup excluded any hip strategy. Any postural control effort was applied through the ankles. Another more simple explanation would relate to the positive correlation between ankle joint torques and gravity. As with any passive, spring-like stabilizing mechanism, increasing torque would lead to increasing body excursions. As a partial compensation, rapid strong reflex-induced postural reactions in distal muscles (Masani et al., 2013), and co-contraction (Bruhn et al., 2004; Hortobágyi et al., 2009; Nagai et al., 2011; Sayenko et al., 2012) may contribute to the frequency rise. Varying inertia and damping did not affect sway amplitude or velocity to a significant degree.

The relative size of postural reactions as a function of anterior-posterior platform tilts, exemplified by GAIN, positively correlated with the amount of gravity applied. This is again in line with the larger ankle torque leading to larger body excursions, as mentioned in the previous paragraph (Ritzmann et al., 2015). Damping worked as a velocity-related resistance against the movement of the foot plate. This behavior reduces excursions just like ankle rigidity would do. Consequently, increasing damping reduced GAIN. The effect of inertia on GAIN did not reach statistical significance. That may be, in part, due to the low values of the imposed inertia effect which was related to the technical feasibility. Because we technically had to feed back the second derivative (acceleration) of the platform position as a force signal, this signal contained a large degree of high frequency noise, which limited the stability of the whole platform system. Higher acceleration gains led to platform oscillations that were perceived by the subject and might have been able to bias the outcome. The stimulus size determined the GAIN in terms of a negative correlation. The larger the stimulus size the lower the relative postural reactions. This closely resembles the relationships in free standing on a moving platform (Peterka, 2002; Engelhart et al., 2014). The temporal relationship between stimulus and response as represented by PHASE, varied across stimulus conditions. However, the amount of the effects was small. Apart from the effect of stimulus amplitude, mean PHASE shifts varied between 4° and 32°. Stimulus amplitude positively correlated with PHASE with a range from 14° to 70°. This may be due to the change of postural strategy with larger stimuli, as, again, is also seen in free standing, and reported below.

In general, the virtual balance task presented here bear resemblance to free standing. Spontaneous sway measures such as sway amplitude (RMS) or sway velocity (MV) were larger in the virtual balance task. On a first look, the frequency distribution of postural reactions seemed to be similar. The similarity covers the GAIN dependency on stimulus frequency with a maximum GAIN around 0.3 Hz in both virtual balancing and free stance: With increasing frequency GAIN values decreased in both cases. Moreover, the non-linearity of postural responses as a function of stimulus size in terms of a GAIN reduction with larger stimulus sizes was similar. PHASE curves were similar in that low stimulus frequencies induced a small PHASE delay while high frequencies induced a large PHASE delay. Finally, the measure for reproducibility of the postural response, i.e., coherence, was similar.

In more detail, GAIN values of virtual balancing where larger than those of free stance and PHASE values of virtual balancing display a flattened profile as a function of frequency. Spontaneous sway and perturbed stance features of virtual balancing resemble abnormalities of vestibular loss patients (Maurer et al., 2006; Goodworth and Peterka, 2010). In fact, space cues (i.e., visual and vestibular feedback) are limited in the virtual balancing task due to the experimental setting. As the body is leaning against the backboard, the vestibular and visual systems sense zero movement, while the proprioceptive system detects the ankle angle representing the virtual body orientation. As such, vestibular and visual systems do not contribute to virtual balancing, which may impair an even closer resemblance to free standing. In future experiments, we will add visual contributions by displaying a virtual visual background according to the virtual body movements. Another limitation for the comparison between real stance and virtual balancing might have been that subjects' sway during real stance could have been affected by intermittent touch of the safety ropes which, in principle, have the potential to reduce sway as an additional space reference (Wing et al., 2011). Therefore, we aimed to avoid in our experimental setup that ropes ever touch the subject. The ropes, which served as a replacement for a body harness, were fixed to the ceiling at about 30 cm in forward direction with respect to the foot position of the subjects. We monitored the subjects during the experiments and touch did not occur.

When comparing our results during unperturbed and perturbed free stance to own earlier studies and to the literature (Prieto et al., 1996; Peterka, 2002; Maurer and Peterka, 2005; Maurer et al., 2006; Goodworth and Peterka, 2010; van der Kooij and Peterka, 2011; Engelhart et al., 2014; Wiesmeier et al., 2015) we did not find any major differences.

When adjusting the parameters of virtual balancing, i.e., stimulus amplitude, virtual gravity, virtual damping, and virtual inertia, to optimally resemble transfer functions of postural reactions of free standing, we found a large stimulus amplitude, low gravity and large damping values. However, our subjects felt maximal resemblance between virtual balancing and free standing at low stimulus amplitude, low gravity, and without any damping and inertia.

This discrepancy may, again, point to additional differences in the experimental settings of virtual vs. free balancing. During virtual balancing, subjects did not move their own body. Possible postural reactions were confined to the ankle joint. Even if the major postural reactions originate from the ankle joint in free standing, (Horak et al., 1989), the hip joint usually contributes to postural reactions, especially in the high frequency range. The virtual balancing task may, therefore, still elicit certain changes in the balancing strategy. Moreover, discrepancies between virtual balancing and free standing might be explained by the absence of space cues (see above).

Patients' benefit of virtual balancing may rely on the potentially increased mobility in a reduced gravity environment. Moreover, balancing could be trained without the threat to fall. Patient groups, who may benefit from such training, may include those who are not able to support their complete body weight like, e.g., hemiparetic/paraparetic patients or patients suffering from axial fractures. In this study we were able to show the feasibility of a virtual balancing paradigm. In a future version, the apparatus could be integrated into boots and may act as an exoskeleton around the ankle angle. The main purpose of such a training prosthesis is to rehearse balance, which is the major prerequisite for free walking. Further studies are needed to show that practicing virtual balance affects free stance, and to evaluate which patients may benefit from such a training.

## Author contributions

DB was responsible for data acquisition, analysis, and interpretation and contributed to writing the first draft. DD contributed to the conception of the work, to data analysis, and interpretation. IW contributed to data interpretation, drafted and edited the final manuscript for submission and revised the work critically. CM was responsible for the conception of the work, for data analysis and interpretation and wrote the first draft. All authors approved the final manuscript.

### Conflict of interest statement

The authors declare that the research was conducted in the absence of any commercial or financial relationships that could be construed as a potential conflict of interest.
